# Derivation of neural crest stem cells from human epidermal keratinocytes requires FGF‐2, IGF‐1, and inhibition of TGF‐β1

**DOI:** 10.1002/btm2.10109

**Published:** 2018-10-01

**Authors:** Georgios Tseropoulos, Samaneh Moghadasi Boroujeni, Vivek K. Bajpai, Pedro Lei, Stelios T. Andreadis

**Affiliations:** ^1^ Dept. of Chemical and Biological Engineering University at Buffalo Buffalo NY 14260; ^2^ Dept. of Biomedical Engineering University at Buffalo Buffalo NY 14228; ^3^ Center of Excellence in Bioinformatics and Life Sciences Buffalo NY 14203

**Keywords:** epidermal keratinocytes, neural crest, soluble signals

## Abstract

Neural crest (NC) cells play a central role in forming the peripheral nervous system, the craniofacial skeleton, and the pigmentation of the skin during development due to their broad multilineage differentiation potential into neurons, Schwann cells, melanocytes, and mesenchymal stem cells. Recently, we identified an easily accessible source of pluripotent NC stem cells from human inter‐follicular keratinocyte (KC) cultures (KC‐NC). In this work, we examined specific conditions for the derivation of NC from KC cultures. More specifically, we examined the role of two growth factors, FGF2 and IGF1, in NC proliferation and in expression of two potent NC transcription factors, Sox10 and FoxD3. Using specific chemical inhibitors, we uncovered that the downstream regulatory pathways AKT/PI3K, MEK/ERK, and JNK/cJun may be critical in Sox10 and FoxD3 regulation in KC‐NC. The TGF‐β1 pathway was also implicated in suppressing Sox10 expression and NC proliferation. In summary, our study shed light into the role of FGF2, IGF1, and TGF‐β1 on the induction of NC from KC cultures and the pathways that regulate Sox10 and FoxD3. We also established culture conditions for sustaining KC‐NC multipotency and, therefore, the potential of these cells for regenerative medicine and cellular therapies.

## 1 INTRODUCTION

Neural crest (NC) is an embryonic structure, unique to vertebrates, at the junction of neural and non‐neural ectoderm, from which NC cells arise and migrate laterally along the length of developing neural tube. During the process of gastrulation, the growing embryo is divided into the three germ layers: ectoderm, mesoderm, and endoderm. The ectoderm undergoes subsequent division, forming the neural and non‐neural ectoderm. The region separating these two is commonly referred to as the neural plate border and is the origin of NC stem cells in embryogenesis.^1^ The NC is a transient cell population that is capable of self‐renewal and possesses multipotency. After the neural‐ectoderm folds to form the neural tube, NC stem cells develop a migratory phenotype and delaminate from its dorsal aspect.^2^ This mobility is made possible by the ability of NC cells to undergo epithelial to mesenchymal transition (EMT), enabling migration throughout the developing embryo, where they contribute to portions of the craniofacial skeleton, neurons, peripheral glia, cardiac outflow tract, smooth muscle cells, chondrocytes, adipocytes, melanocytes, and so forth.^1^ This was also confirmed through the use of in vitro clonal cultures, where individual NC cells were isolated, expanded, and subjected to factors known to induce differentiation.^3^

Regulation of such a transient population of cells requires a complex network of signaling pathways to maintain the stem cell‐like state and control all subsequent cellular changes. There are three pathways that are mainly reported to have a greater significance than others during NC induction: FGF, Wnt, and BMP signaling.^4^ These pathways lead to the expression of NC specifiers that define the NC state, albeit transiently, from the beginning to the end of induction. Some of these specifiers include among others, the SoxE family of factors as well as Pax3 and Zic1.^5^, ^6^, ^7^, ^8^ While the importance of FGF signaling in the NC induction has been postulated in a number of studies, mainly focusing on the *Xenopus* embryo model,^9^, ^10^, ^11^, ^12^ further investigation is needed to expand these findings in NC stem cells isolated from adult humans.

Genetic mutations can result in dysregulated NC development leading to many congenital human diseases, such as cardiovascular defects and craniofacial abnormities, collectively known as neurocristopathies,^13^ myelopathies, neural degenerative diseases, and so forth. Therefore, cultures of human NC cells can provide a model to study human disease and a source of stem cells for treatment of neurodegenerative diseases that may be currently hindered by the lack of an easily accessible and autologous cell source. Interestingly, recent studies have successfully isolated NC cells from different tissues in the adult body, including the adult hair follicle, craniofacial sources such as the palate and the oral mucosa.^14^, ^15^, ^16^, ^17^ Recently, our laboratory showed that NC could be derived from cultures of epidermal KCs isolated from glabrous neonatal foreskin. KC‐derived NC could be coaxed to differentiate into functional neurons, Schwann cells, melanocytes, osteocytes, chondrocytes, adipocytes and smooth muscle cells, in vitro and in vivo, in lineage tracing experiments in chick embryos.^17^ Given the accessibility of human skin, KC‐derived NC may provide a valuable source of multipotent stem cells for treatment of myelopathies and other debilitating neurodegenerative diseases. Therefore, it is critical to understand the factors affecting NC derivation, including expansion and maintenance of the NC phenotype and multilineage differentiation potential.

In this study, we focused on the role of growth factors and downstream signaling pathways that may be important in derivation of NC from human KC and identified the culture conditions that may be optimal for NC proliferation and expression of key transcription factors, Sox10 and FoxD3, which have been shown to be critical for maintenance of the NC phenotype and the NC multilineage differentiation potential.

## 2 MATERIALS AND METHODS

### 2.1 Isolation of epidermal cells

Glabrous (lacking hair follicles) foreskin from 1‐ to 3‐day‐old neonates was procured from John R. Oishei Children's Hospital, Buffalo. Skin samples were washed three times with PBS, dissected into pieces (~3 × 1 cm), enzymatically digested with dispase II protease (Sigma, St. Louis, MO, USA) for 15‐20 hr at 4 °C. The epidermis was, afterward, separated from the dermis manually using fine forceps. The separated epidermis was then treated with Trypsin‐EDTA (0.25%) (Life Technologies, Carlsbad, CA, USA) for 10‐15 min at 37 °C, filtered through 70 μm cell strainer (BD Biosciences, Franklin Lakes, NJ, USA), centrifuged and plated on a confluent monolayer of growth‐arrested 3T3/J2 mouse fibroblast feeder cells in keratinocyte growth medium (KCM) consisting of a 3:1 mixture of high glucose Dulbecco's Modified Eagle's Medium (DMEM) and Ham's F‐12 medium (Life Technologies) supplemented with 10% (v/v) fetal bovine serum (FBS, Atlanta Biologicals, Flowery Branch, GA, USA), 100 nM cholera toxin (Vibrio Cholerae, Type Inaba 569 B; Millipore, Burlington MA), 5 μg/mL transferrin (Life Technologies), 0.4 μg/mL hydrocortisone (Sigma), 0.13 U/mL insulin (Sigma), 1.4 × 10^−4^ M adenine (Sigma), 2 × 10^−9^ M triiodo‐L‐thyronine thyronine (Sigma), 1× antibiotic‐antimycotic (Life Technologies) and 10 ng/mL epidermal growth factor (EGF, BD Biosciences). The cells were cultured in KCM for 8‐10 days. Afterward, the 3T3/J2 feeder layer was detached after a 10‐min versene treatment. The remaining cells were treated with trypsin–EDTA (0.25%), which was then neutralized by a solution containing 10% FBS in PBS and plated in KC serum free growth medium (KSFM, Epilife medium with Human Keratinocyte Growth Supplement; Life Technologies). Further expansion took place in KSFM prior to NC induction. Passages 1‐3 KC were used in all experiments.

### 2.2 Induction of KC into NC stem cell fate

For induction into the NC fate, KC were cultured at a density of 8‐10 × 10^3^ cells/cm^2^ in collagen type I coated dishes (10 μg collagen type I per cm^2^; BD Biosciences) in the presence of NC induction medium (NCIM), comprising basal medium (EBM‐2 medium; Lonza, Basel, Switzerland) plus 2% (v/v) FBS, 10 μg per ml heparin (Lonza), 100 μg per ml ascorbic acid (Lonza), and 0.5 μg per ml hydrocortisone (Lonza), 1× Gentamicin/Amphotericin‐B (Lonza) and supplemented with 10 ng/mL fibroblast growth factor 2 (FGF2; BD Biosciences) and 10 ng/mL insulin‐like growth factor 1 (IGF1, Lonza). FGF2 and IGF1 concentrations were optimized in previous studies in our lab.^17^ For our signaling pathways investigation, the following inhibitors were used: PD173074 (Cayman Chemical, Ann Arbor, MI, USA, concentration: 1 μM), CH5183284 (Sellechem, Boston, MA, USA, concentration: 0.5 μM), SB431542 (Sigma, concentration: 10 μM). All inhibitors were dissolved in DMSO.

### 2.3 Immunocytochemistry

Cells were washed with cold PBS (4 °C) and permeabilized with 4% (vol/vol) paraformaldehyde (10 min, room temperature; Sigma). Permeabilization (10 min, room temperature) was performed with 0.1% (vol/vol) triton X‐100, (Sigma) in PBS and samples were blocked with 5% (vol/vol) normal goat serum (Life Technologies) in PBS. The cells were incubated with primary antibodies overnight (4 °C) (Supporting Information Table S1) followed by incubation with appropriate secondary antibodies (1 hr, room temperature) conjugated with Alexa 488 or Alexa 594 (Life Technologies). Hoechst 33342 (Thermo Fisher Scientific, Grand Island, NY) was used for nuclear staining. Cells that were incubated with only secondary antibody served as controls.

### 2.4 Imaging and image analysis

Immunocytochemistry images were acquired using a Zeiss Axio Observer Z1 inverted microscope with an ORCA‐ER CCD camera (Hamamatsu, Japan). The images were acquired using fixed exposure time (NES: 200 ms, SOX10: 400 ms, FOXD3: 500 ms). Cell number quantification was performed using NIH ImageJ. The images were converted to eight‐bit. Manual marking and cell counting were performed for NES+, SOX10+, and FOXD3+ cells using the Cell Counter plugin. For each condition, *n* = 3 separate wells were counted. Statistical significance between the groups was analyzed through Student's *t*‐test (paired, two‐tailed) as described previously^18^ and a confidence interval of 95% was chosen.

## 3 RESULTS

### 3.1 NC stem cells derived from keratinocyte cultures

**Figure 1:**
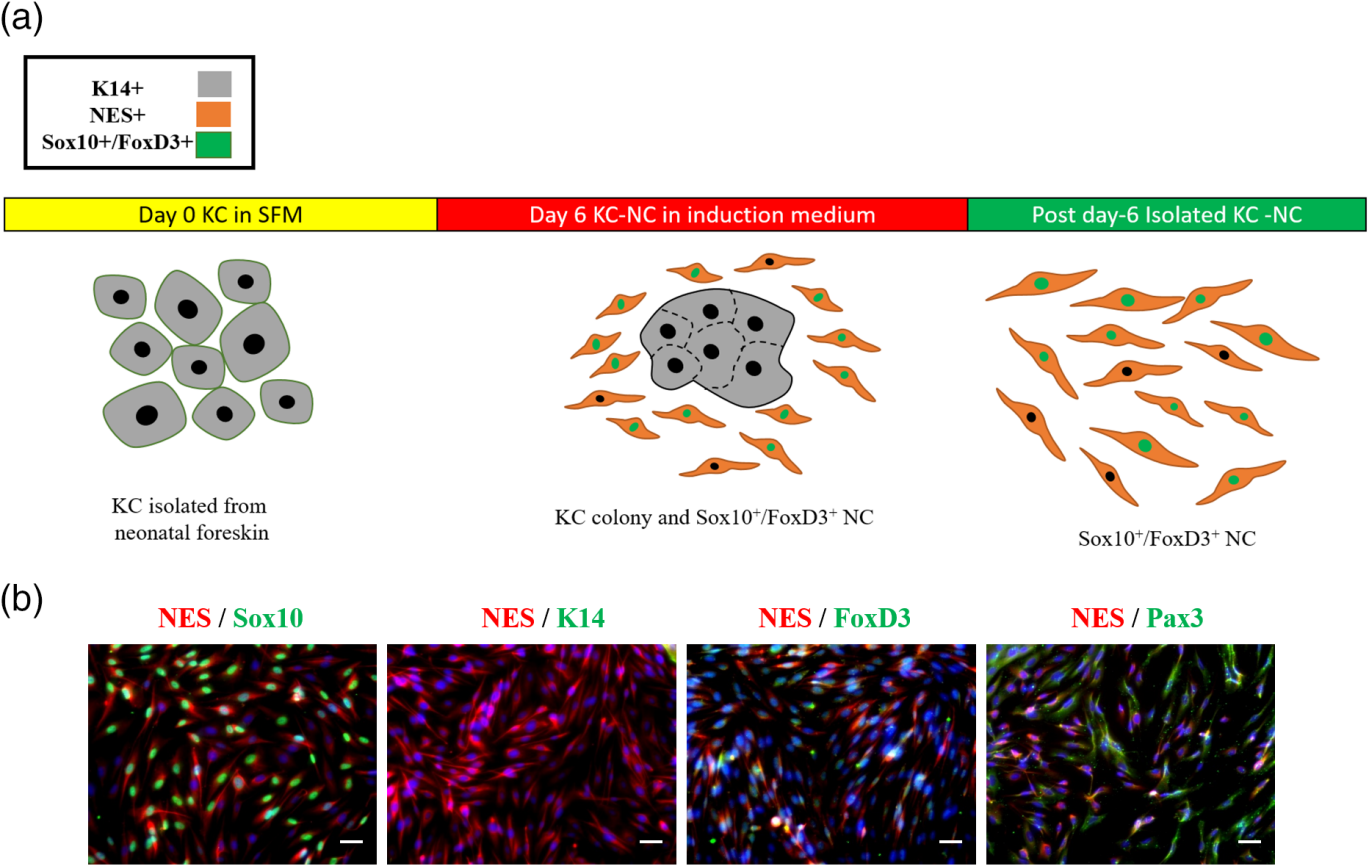
Obtaining NC stem cells from KC cultures. (a) Schematic of the KC‐NC induction process. (b) Induction of pluripotent NCs from KC cultures with medium containing FGF2, IGF1, ascorbic acid, heparin, hydrocortisone, and 2% FBS. After 6 days of induction, the cells express Sox10, FoxD3, NES, PAX3, and lack KC marker K14. Scale bars, 50 μm. Each experiment was repeated three times

An adult NC population has been found in hair follicle's bulge region.^19^ To avoid possible contamination from the NC population present in the bulge region of the hair follicle, we isolated KC from the interfollicular epidermis of glabrous skin from 1 to –3 days old neonates. KC were isolated from three individual donors and cultured in serum‐free, low calcium KSFM, where they were maintained as single epithelial cells expressing keratin 14 (K14) (Figure 1a).

One day after plating, KSFM was changed to the NCIM (EBM2 basal medium containing FGF2, IGF1, ascorbic acid, hydrocortisone, heparin, 2% FBS, and high calcium). Within 24 hr, NCIM caused KC aggregation and after 3‐4 days a number of small NC cells could be observed around the KC colonies. By Day 7, NC cells proliferated yielding a large number of cells surrounding the KC colonies (~30‐40 × 10^2^/10^4^ KC seeded), in agreement with recent results from our laboratory.^17^ Immunostaining showed that KC had formed colonies expressing K14 (not shown), while small, spindle‐shaped cells expressed Nestin (NES)—an intermediate filament protein that is expressed in both central nervous system (CNS) progenitors and NC stem cells^19^, ^20^–as well as other NC markers such as Sox10, FoxD3, and Pax3 but were lacking K14 (Figure 1b**)**.

### 3.2 The effect of induction medium on NC yield from KC cultures

Next, we tested whether the basal medium had an effect on NC induction and proliferation. To this end, we tested three basal media, EBM2, DMEM, and EpiLife. The concentration of calcium in the first two was high (1.8 mM), while in the third was low (0.09 mM). All basal media were supplemented with FGF2, IGF1, ascorbic acid, hydrocortisone, and heparin.

**Figure 2:**
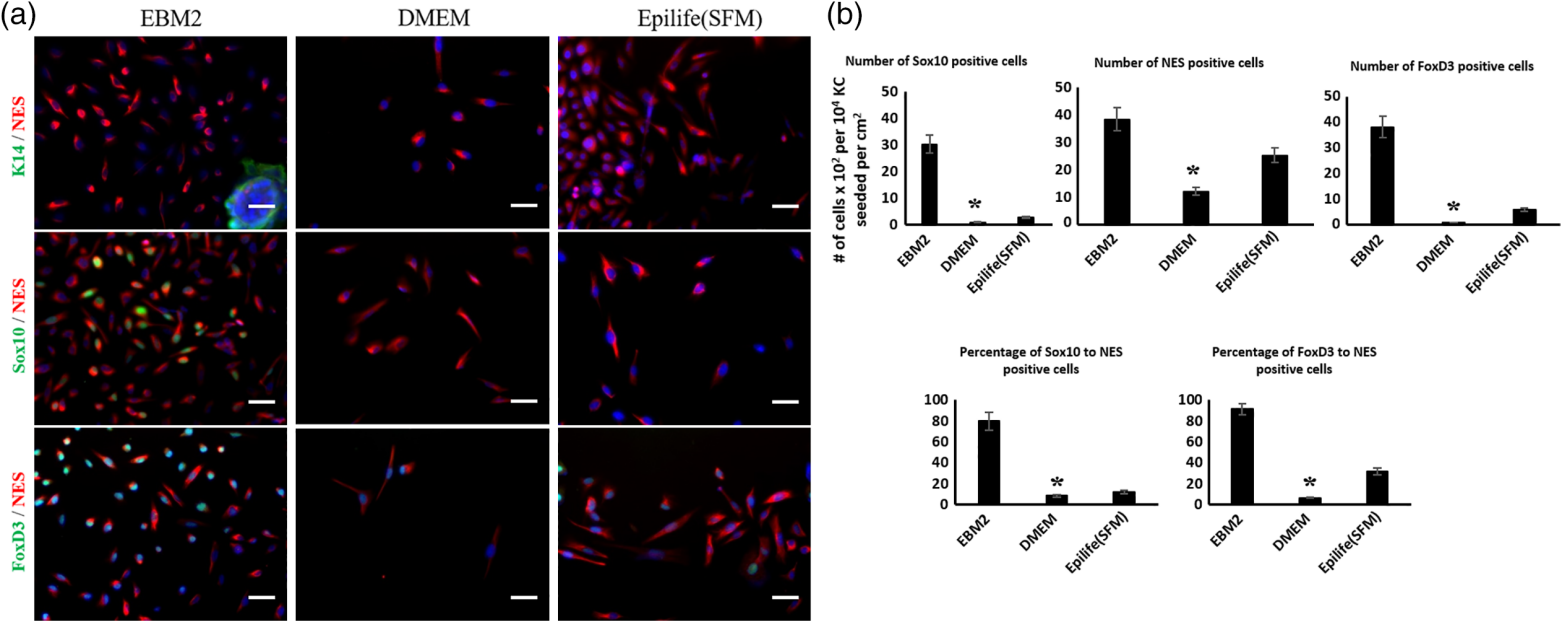
The effect of different basal media (EBM2, DMEM, Epilife‐SFM) in KC‐NC induction. (a) Immunostaining shows expression of Sox10 and FoxD3 only in the EBM2 condition. (b) Quantification of immunofluorescence images using ImageJ. Numbers of Sox10+ and FoxD3+ cells are significantly higher in EBM2 medium. Scale bars, 50 μm. All values are mean ± SD. Each experiment was repeated three times

Interestingly, EBM2 supported NC proliferation to much higher extent as compared to KSFM and DMEM as evidenced by the number of NES^+^/K14^−^ cells after 7 days of induction per 10^4^ KC/cm^2^ initially plated (Figure 2a, b). In addition, the number of cells expressing Sox10 or FoxD3 and the percentage of Sox10+ or FoxD3+ among the NES‐expressing cells were the highest in EBM2 medium and the lowest in DMEM. Finally, the cells grown in DMEM appeared more elongated, resembling a fibroblastic phenotype after 7 days in culture.

**Figure 3:**
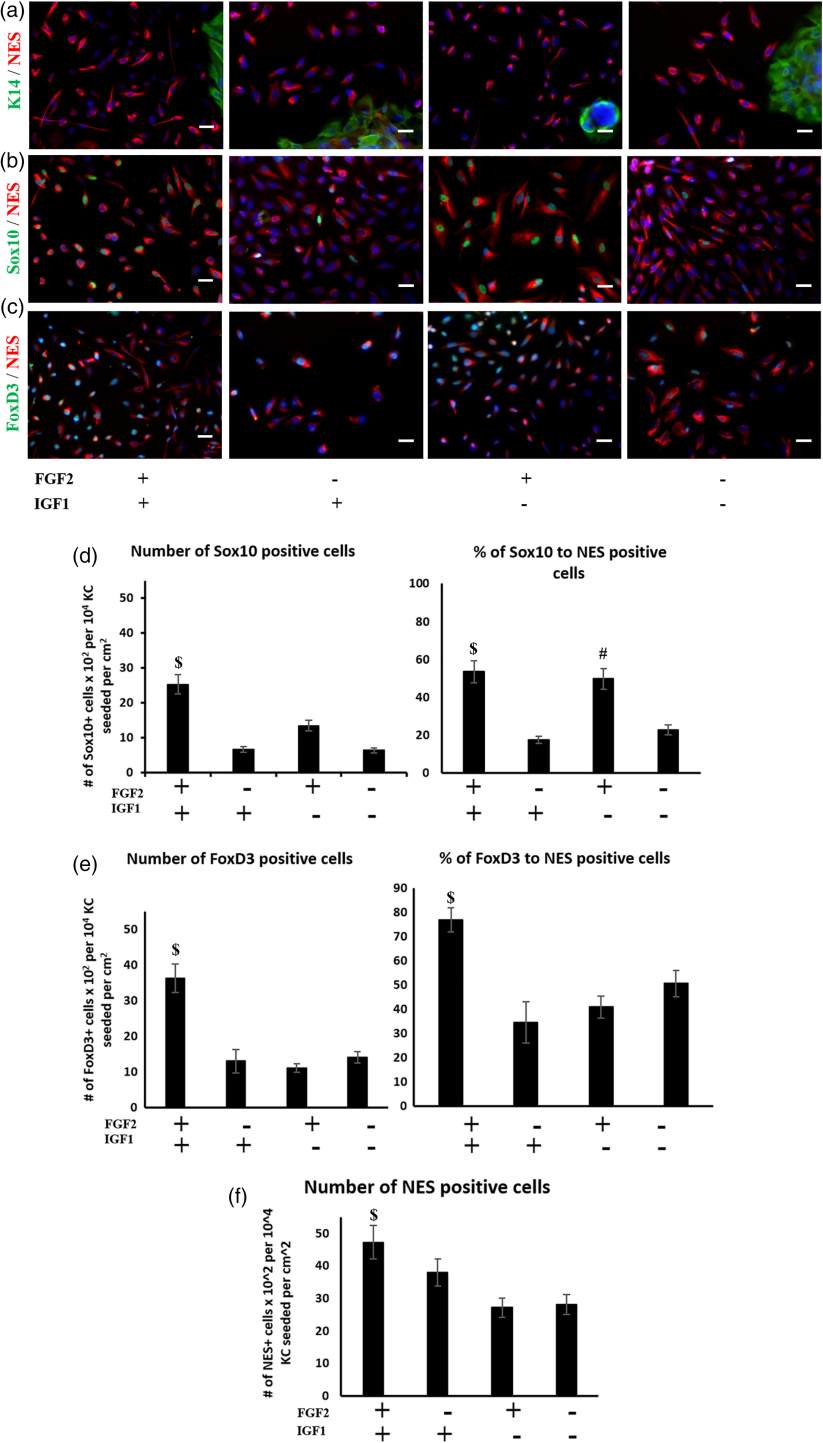
Effect of soluble factors in NC induction. Immunostaining for (a) NES and K14; (b) Sox10 and NES; (c) FoxD3 and NES under the indicated conditions. (d)‐(f) Quantification of the immunocytochemical data using ImageJ. Scale bars, 50 μm. Each experiment was repeated three times

Next, we tested the effect of FGF2 and IGF1 in NCIM using immunostaining for the markers NES, Sox10 and FoxD3 (Figure 3a‐c). Quantification of the number of NES, Sox10, and FoxD3 positive cells, as well as the fraction of Sox10+ or FoxD3+ within the NES+ cells was performed using ImageJ. Interestingly, either FGF2 or IGF1 induced proliferation of NC cells, but their combination increased NES+ cells even further, suggesting a synergistic effect. In addition, the fraction of FoxD3+/NES+ cells was similar with either FGF2 (42 ± 3.8, *n* = 3) or IGF1 (35 ± 2.6%, *n* = 3) but increased dramatically when both were present (91 ± 10.3%, *n* = 3). However, the fraction of Sox10+/NES+ cells was dependent primarily on FGF2 (53 ± 7.7%, *n* = 3), as IGF1 alone yielded a low fraction (18 ± 2.3%, *n* = 3) and their combination did not show significantly better results (56 ± 4.9%, *n* = 3) than FGF2 alone (Figure 3d‐f). Therefore, the total number of NES+ and the fraction of FoxD3 depended on both FGF2 and IGF1 but Sox10 expression depended mostly on FGF2.

**Figure 4:**
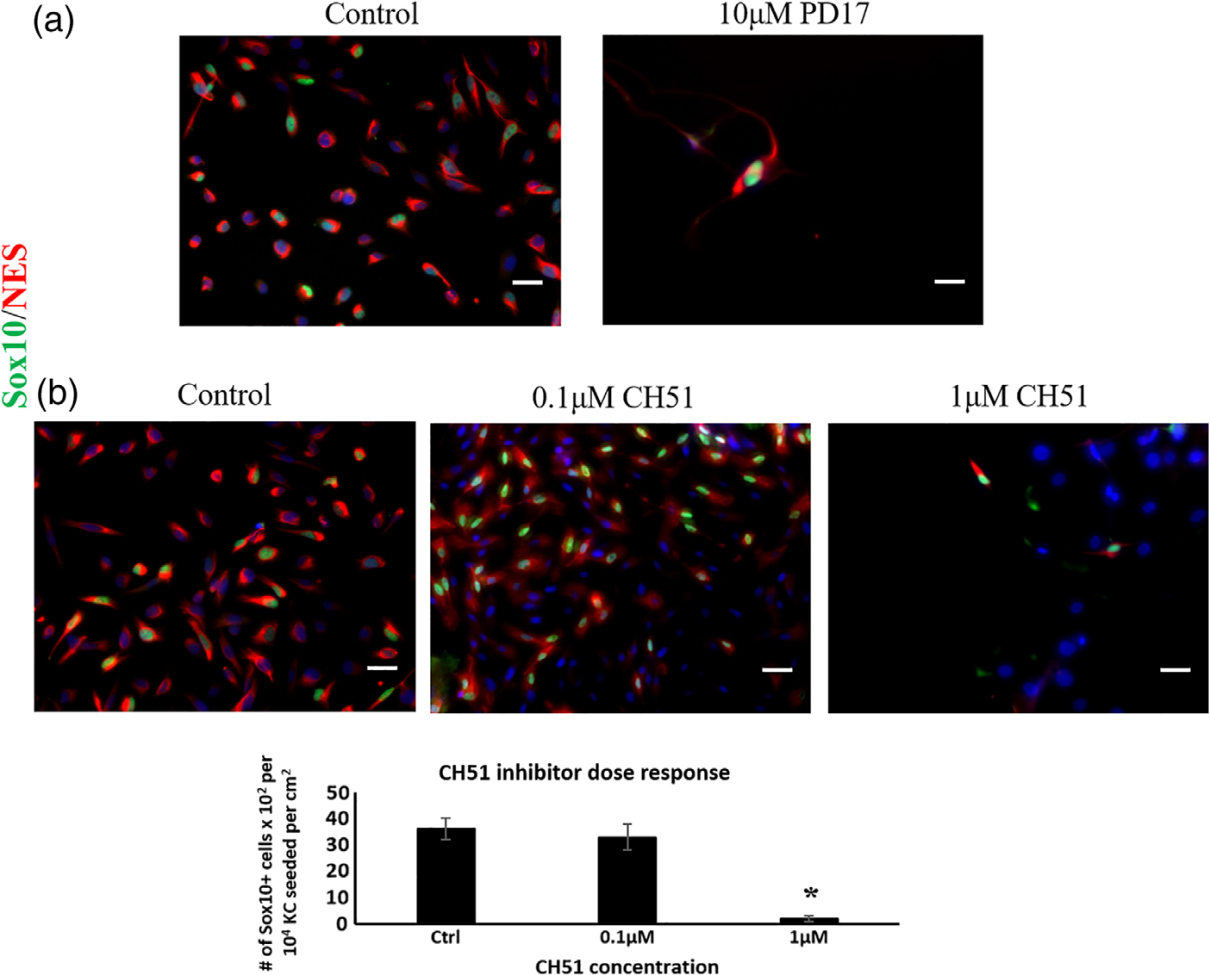
The role of FGF2 in the proliferation of NC from KC cultures. (a) Treatment with the tyrosine kinase inhibitor PD173074 (10 μM) inhibited the proliferation of NC completely. (b) Exposing the cultures to high concentration of FGF inhibitor CH51 (1 μM) fails to yield NC after 7 days of induction. Scale bars, 50 μm. Each experiment was repeated three times

Notably, as shown by the percentage of Sox10+/NES+ cells, the presence of FGF2 was sufficient for Sox10 expression even in the absence of serum (61 ± 5.2%, *n* = 3), but IGF1 was not (7.6 ± 3.3%, *n* = 3). As Sox10 is considered a key transcription factor of the NC lineage, which is also necessary for differentiation towards neurons and Schwann cells,^1^ we investigated the effect of FGF2 signaling on NC induction. Indeed, blocking fibroblast growth factor receptor (FGFR) with either PD173074 or CH5183284 chemical inhibitors diminished the number of NES+ cells and the percentage of Sox10+/NES+ cells after 7 days of induction (Figure 4a, b**)**.

### 3.3 Akt and Erk1/2 but not Rac are necessary for NC induction

**Figure 5:**
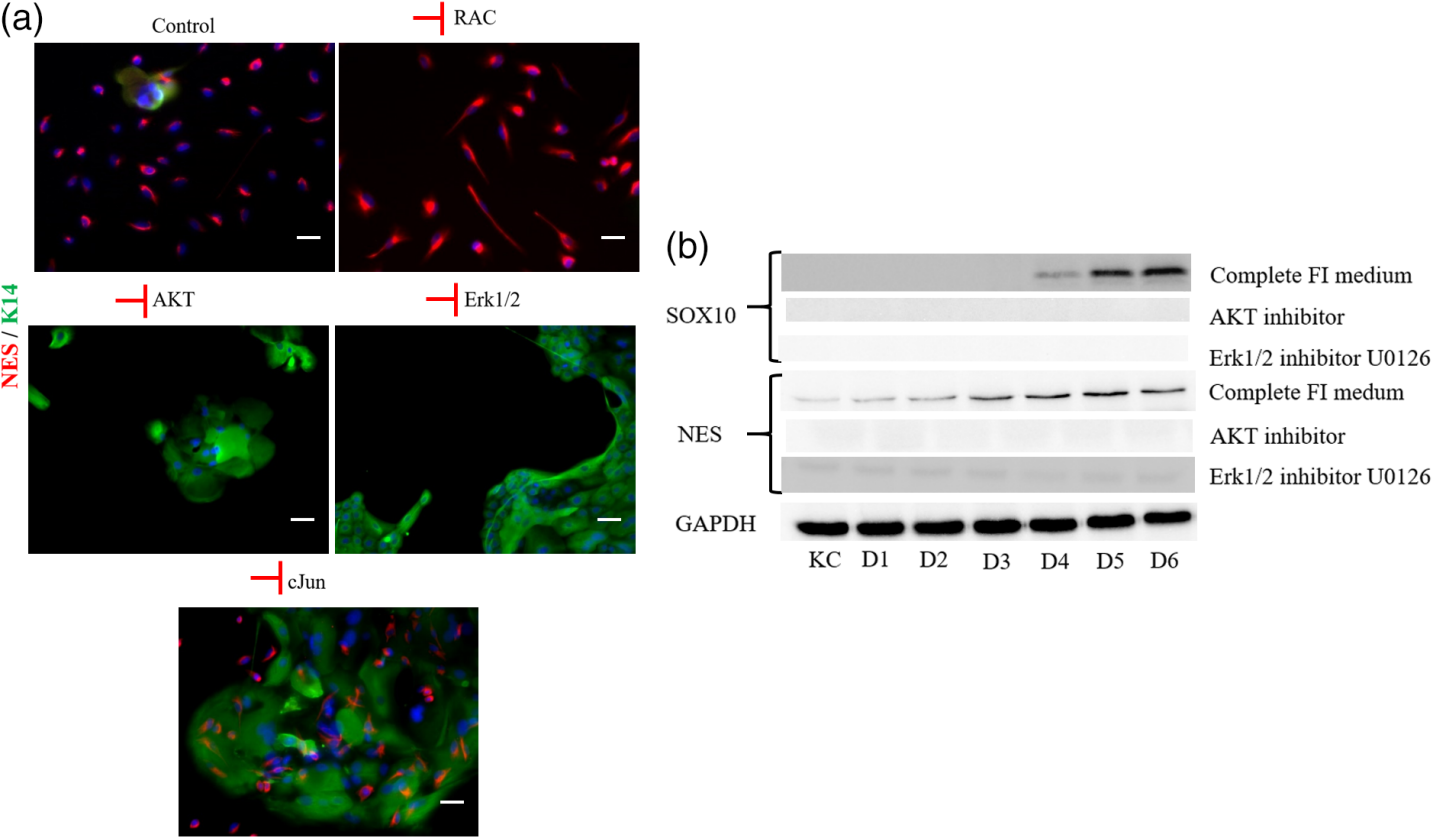
Investigation of intracellular pathways in NC induction. KC were induced with NCIM in the presence of inhibitors of the indicated pathways. (a) Immunostaining for NES and K14 on Day 7 of induction. (b) Western blot showing no expression of Sox10 and NES, when the cultures were exposed to Akt or Erk1/2 inhibitor. Scale bars, 50 μm. Each experiment was repeated three times

Next, we investigated which of the pathways downstream the FGFR may play a pivotal role in NC induction. To this end, we inhibited the Rac, Akt, Erk1/2, and JNK at the start of the induction. Inhibiting Akt or Erk1/2 diminished the number of NES+ cells, while Rac inhibition showed no significant effect (Figure 5a). Interestingly, blocking JNK did not eliminate NC induction but restricted NES+ cells within the colonies of K14+ KC.

Western blot analysis (WB) for NES and Sox10 showed that NES was expressed early in the process from Day 1, while Sox 10 appeared much later, on Day 4. Inhibition of either Akt or Erk1/2 eliminated expression of both proteins as shown by immunostaining, suggesting that these two pathways were necessary for induction of NC from cultures of epidermal KC.

### 3.4 Inhibition of TGF‐β pathway increases the number of Sox10+ and FoxD3 positive cells

**Figure 6:**
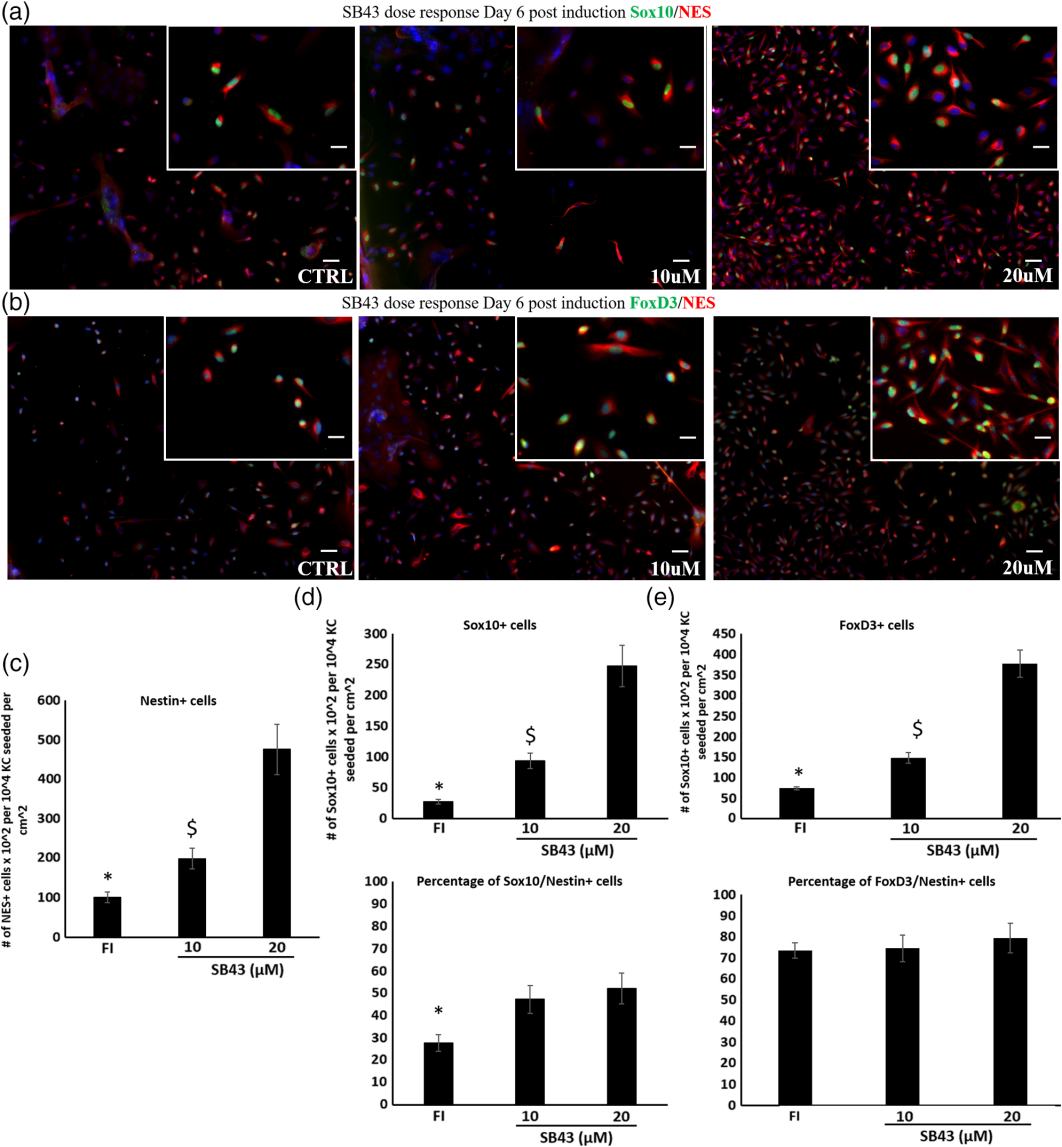
Inhibition of TGF‐β pathway promoted NC induction. KC were induced with NCIM in the presence of SB431542 at 10 or 20 μM. Immunostaining for (a) Sox10 and NES; (b) FoxD3 and NES. (c) The number of NES+ cells in the presence of 10 μM or 20 μM SB43. (d) Percentage of Sox10+ cells in the NES+ population in the presence of 10μM or 20μM SB43. (e) Percentage of FoxD3+ cells in the NES+ population in the presence of 10μM or 20μM SB43. Scale bars, 200 μm. Insets at higher magnification images, scale bars, 50 μm. Each experiment was repeated three times

Previous studies showed that transforming growth factor β1 (TGF‐β1) suppressed Sox10 expression in mouse NC stem cells, suppressing neural and conferring mesenchymal differentiation potential to NC cells in vitro.^21^, ^22^ This result prompted us to hypothesize that inhibition of the TGF‐β1 pathway might promote higher levels of Sox10 expression in human KC‐derived NC cells. To this end, we used the TGF‐β1 receptor (ALK4,5,7) inhibitor SB431542 (SB43) during the KC‐NC induction. During the 6‐day induction period, SB43 increased the number of NES+ cells significantly (~fivefold), suggesting that TGF‐β1 may be suppressing KC‐NC proliferation (Figure 6a‐c). In addition, the percentage of Sox10+/NES+ cells increased significantly, especially at 20 μM SB43 (~twofold, *p* < 0.05, *n* = 3) (Figure 6d), but the percentage of FoxD3+/NES+ cells remained similar to control cells (no SB43) (Figure 6e**)**, suggesting that TGF‐β1 might be suppressing expression of Sox10 but not FoxD3.

### 3.5 Dynamics of NC induction

**Figure 7:**
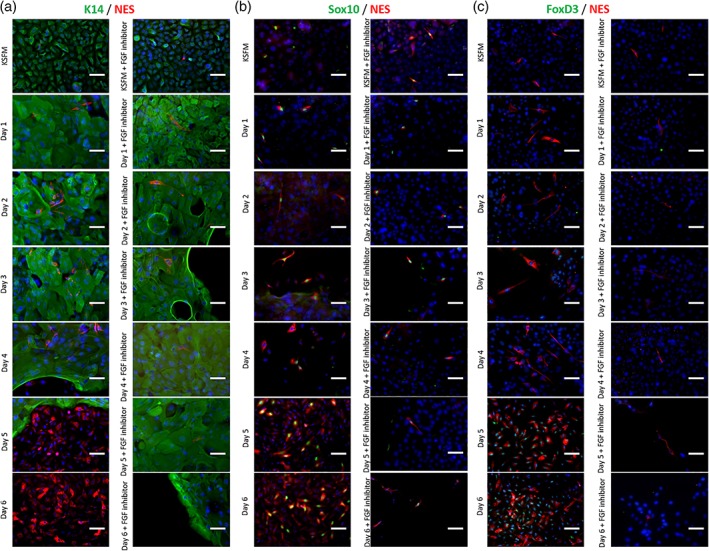
Dynamics of NC induction from KC cultures. (a) Immunostaining for NES/K14 over time showing a small number of NES+ cells surrounding the K14+ colonies. (b) Immunostaining for Sox10/NES over time. (c) Immunostaining for FoxD3/NES over time. Scale bars, 50 μm. Each experiment was repeated three times

To examine the dynamics of NC induction, we followed the expression of Sox10, FoxD3, and NES with immunostaining daily until Day 7 and the cell numbers were normalized to the number of KC that were initially plated (10^4^ cells/cm^2^). After 1 day of induction, KC started to aggregate forming K14 positive colonies and few NES+ cells started to appear within and around the colonies of K14+ cells (Figure 7a,b). The number of NES+ cells increased with time throughout the induction period with population doubling time of 22 ± 2.1 hr (*n* = 3). The number of Sox10+/NES+ cells also increased resulting in 60–70% (*n* = 3) of Sox10+ cells in the NES+ cell population (Figure 7e). Expression of FoxD3 started after Day 4 of induction and the percentage of FoxD3+/NES+ cells reached ~60% (*n* = 3) after 6 days. Treatment with the FGF specific inhibitor CH5183284, eliminated the number of NES+ cells and expression of Sox10 and FoxD3, further suggesting that FGF2 was necessary for NC induction.

## 4 DISCUSSION

Stem cell therapies require large numbers of stem cells, which necessitates the development of conditions that achieve stem cell expansion while maintaining multipotency. Here, we addressed this challenge in the context of NC stem cells and report on the culture conditions under which NC cells can be derived from primary epidermal KC isolated from neonatal glabrous foreskin lacking hair follicles. KC‐derived NC cells (KC‐NC) are small, spindle‐shaped cells that emerge from the KC cultures after 6‐8 days of induction in the presence of FGF2 and IGF1 in EBM2 basal medium. These cells express the filament protein NES as well as NC specific transcription factors Sox10 and FoxD3. Under optimal conditions that is, in the presence of both FGF2 and IGF1 and in the absence of serum around 80% of the cells are positive for Sox10 and FoxD3. Interestingly, the presence of serum reduces the fraction of Sox10+/NES+ cells to less than 60% but maintains the same level of FoxD3+/NES+ cells, suggesting that serum may contain inhibitors of Sox10 expression. Alternatively, serum may promote commitment of NC cells to neuronal fate, which may be accompanied by downregulation of Sox10 expression.^23^, ^24^ It would be interesting to address these two hypotheses and identify the pathways that may contribute to maintenance of the NC phenotype so as to enable long‐term expansion and use of these cells for regenerative medicine.

Our results clearly showed that FGF2 was sufficient to induce Sox10 expression even in the absence of serum, to the same extent as the complete medium. FGF2 was also necessary, as blocking FGFR signaling completely eliminated Sox10 and NES expression as well as NC induction altogether. These results are in agreement with studies that used FGF2 to expand NC cells from hair follicles^25^ or embryoid bodies.^26^ Others implicated FGFR1 in NC migration from the neural plate border area of *Xenopus* embryos^27^ and more recently FGFR4 was also implicated in NC development.^28^

**Figure 8:**
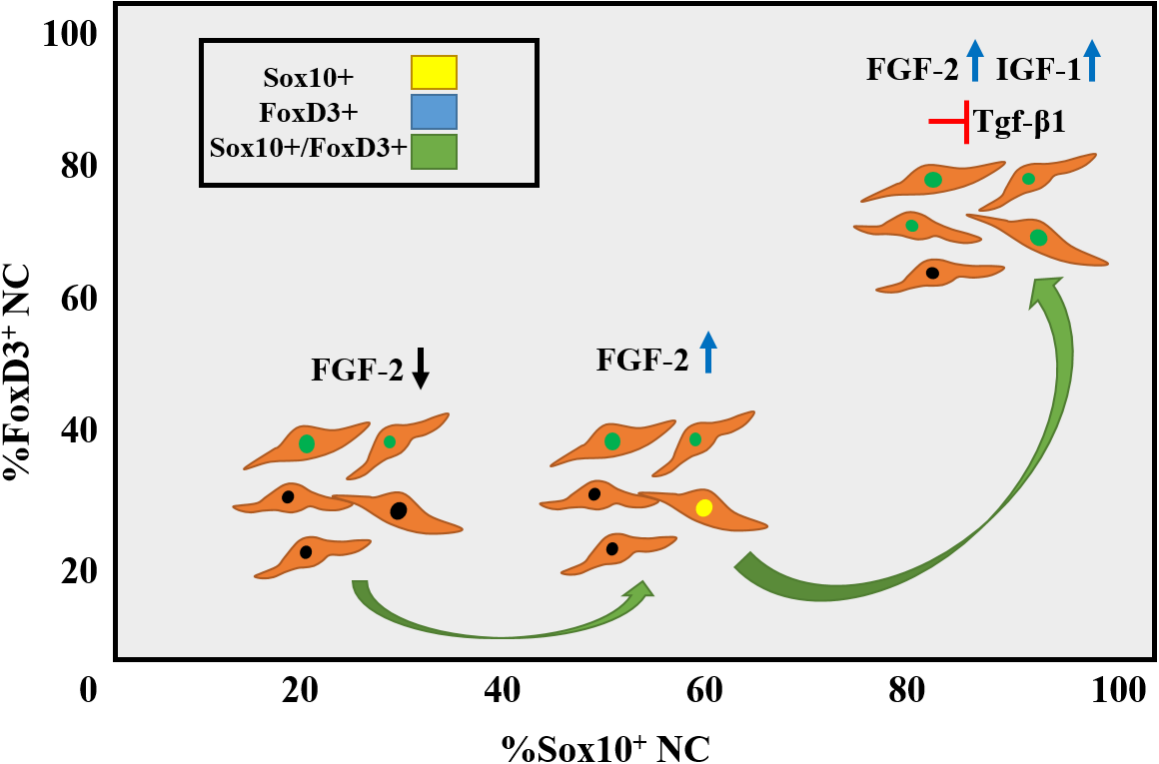
Schematic summarizing the effects of FGF2, IGF‐1, and TGF‐β on Sox10 and FoxD3 expression during NC induction from cultures of human KC

However, IGF1 alone was unable to induce Sox10 expression, especially in the absence of serum. However, in combination with FGF2, it increased the percentage of Sox10+ cells and the total number of NES+ cells, suggesting that IGF1 may have promoted proliferation of NC cells. In addition, IGF1 and FGF2 contributed almost to the same extent in FoxD3 expression. Interestingly, their combined action yielded higher percentage of FoxD3+/NES+ cells than the sum of each growth factor alone. This was especially true in the absence of serum, where each factor was unable to induce FoxD3 expression but when added together they resulted in FoxD3 expression in almost all NES+ cells (Figure 8). This is an interesting result that may suggest that the combination of FGF2 and IGF1 may act cooperatively to induce FoxD3 expression, possibly by activating more than one pathway.

Indeed, we identified that at least two such pathways, PI3K/Akt and MAPK/Erk1/2, were necessary for NC induction. Blocking either pathway completely eliminated NC induction as evidenced by immunostaining for NES. While KC were present in colonies, no NES‐expressing cells were found when either pathway was inhibited. Furthermore, WB showed that neither NES nor Sox10 was expressed when PI3K/Akt or MAPK/Erk1/2 pathway was blocked. The effect of Akt pathway in pluripotency has been recently discussed in the context of embryonic and progenitor cell differentiation patterns.^29^ Furthermore, activating MEK was shown to prolong the expression of pluripotency marker Sox3,^28^ a Sox family member that is also regulated by FGF signaling.^30^ On the other hand, inhibition of Rac had no effect on the number of NES+ cells after 8 days of induction. It will be interesting to examine whether the PI3K/Akt and MAPK/Erk1/2 pathways are also important in the maintenance of the NC phenotype and/or the differentiation of NC stem cells to NC‐specific lineages such as neurons or Schwann cells. Finally, inhibition of TGF‐β1 pathway appeared to have a positive effect on the proliferation of NES+ cells and the fraction of Sox10+ cells, suggesting that TGF‐β1 might be suppressing Sox10 expression and NES+ cell proliferation.

Previously, we showed that KC‐NC are multipotent stem cells that can be coaxed to differentiate into neurons, Schwann cells, melanocytes, and mesenchymal stem cell derivatives (osteocytes, chondrocytes, adipocytes, and smooth muscle cells). Most notably, upon transplantation into chick embryos, KC‐NC migrated along stereotypical pathways and gave rise to multiple NC derivatives, providing strong support of their NC phenotype.^17^ Given the accessibility of human skin and the high proliferation capacity of KC and KC‐NC, these cells represent a potentially useful source of multipotent stem cells for treatment of demyelinating diseases or spinal cord injuries. They could also be used to study diseases of the central or peripheral nervous system for example, neurocristopathies,^31^, ^32^ similar to human induced pluripotent stem cells but without the need for reprogramming to the pluripotent state. This readily accessible source of NC cells may have significant impact on regenerative medicine, understanding human disease and facilitating drug discovery.

## Supporting information

Supplementary Table 1

## References

[1] Sauka‐Spengler T , Bronner‐Fraser M . A gene regulatory network orchestrates neural crest formation. Nat Rev Mol Cell Biol. 2008;9(7):557‐568. 18523435 10.1038/nrm2428

[2] Saadai P , Wang A , Nout YS , et al. Human induced pluripotent stem cell‐derived neural crest stem cells integrate into the injured spinal cord in the fetal lamb model of myelomeningocele. J Pediatr Surg. 2013;48(1):158‐163. 23331809 10.1016/j.jpedsurg.2012.10.034

[3] Dupin E , Sommer L . Neural crest progenitors and stem cells: from early development to adulthood. Dev Biol. 2012;366(1):83‐95. 22425619 10.1016/j.ydbio.2012.02.035

[4] Prasad MS , Sauka‐Spengler T , LaBonne C . Induction of the neural crest state: control of stem cell attributes by gene regulatory, post‐transcriptional and epigenetic interactions. Dev Biol. 2012;366(1):10‐21. 22583479 10.1016/j.ydbio.2012.03.014 PMC3354335

[5] LaBonne C , Bronner‐Fraser M . Neural crest induction in Xenopus: evidence for a two‐signal model. Development. 1998;125(13):2403‐2414. 9609823 10.1242/dev.125.13.2403

[6] Mayor R , Morgan R , Sargent MG . Induction of the prospective neural crest of Xenopus. Development. 1995;121(3):767‐777. 7720581 10.1242/dev.121.3.767

[7] Mayor R , Guerrero N , Martınez C . Role of FGF and Nogginin neural crest induction. Dev Biol. 1997;189(1):1‐12. 9281332 10.1006/dbio.1997.8634

[8] Sauka‐Spengler T , Meulemans D , Jones M , Bronner‐Fraser M . Ancient evolutionary origin of the neural crest gene regulatory network. Dev Cell. 2007;13(3):405‐420. 17765683 10.1016/j.devcel.2007.08.005

[9] Launay C , Fromentoux V , Shi D‐L , Boucaut J‐C . A truncated FGF receptor blocks neural induction by endogenous Xenopus inducers. Development. 1996;122(3):869‐880. 8631265 10.1242/dev.122.3.869

[10] Shi D‐L , Launay C , Fromentoux V , Feige J‐J , Boucaut J‐C . Expression of fibroblast growth factor receptor‐2 splice variants is developmentally and tissue‐specifically regulated in the amphibian embryo. Dev Biol. 1994;164(1):173‐182. 8026621 10.1006/dbio.1994.1189

[11] Kengaku M , Okamoto H . bFGF as a possible morphogen for the anteroposterior axis of the central nervous system in Xenopus. Development. 1995;121(9):3121‐3130. 7555736 10.1242/dev.121.9.3121

[12] Lamb TM , Harland RM . Fibroblast growth factor is a direct neural inducer, which combined with noggin generates anterior–posterior neural pattern. Development. 1995;121(11):3627‐3636. 8582276 10.1242/dev.121.11.3627

[13] Theveneau E , Mayor R . Neural crest determination and migration Principles of Developmental Genetics. 2nd ed. Moody S. A. (Ed.); New York: Academic Press, 2014:315‐330.

[14] Kaltschmidt B , Kaltschmidt C , Widera D . Adult craniofacial stem cells: sources and relation to the neural crest. Stem Cell Rev Rep. 2012;8(3):658‐671. 10.1007/s12015-011-9340-9 22170630

[15] Takahashi C , Yoshida H , Komine A , Nakao K , Tsuji T , Tomooka Y . Newly established cell lines from mouse oral epithelium regenerate teeth when combined with dental mesenchyme. In Vitro Cell Dev Biol Anim. 2010;46(5):457‐468. 20033791 10.1007/s11626-009-9265-7 PMC2862945

[16] Marynka‐Kalmani K , Treves S , Yafee M , et al. The lamina propria of adult human oral mucosa harbors a novel stem cell population. Stem Cells. 2010;28(5):984‐995. 20474080 10.1002/stem.425

[17] Bajpai VK , Kerosuo L , Tseropoulos G , et al. Reprogramming postnatal human epidermal keratinocytes toward functional neural crest fates. Stem Cells. 2017;35(5):1402‐1415. 28142205 10.1002/stem.2583 PMC5543412

[18] Burgess A , Vigneron S , Brioudes E , Labbé J‐C , Lorca T , Castro A . Loss of human Greatwall results in G2 arrest and multiple mitotic defects due to deregulation of the cyclin B‐Cdc2/PP2A balance. Proc Natl Acad Sci. 2010;107(28):12564‐12569. 20538976 10.1073/pnas.0914191107 PMC2906566

[19] Sieber‐Blum M , Grim M , Hu Y , Szeder V . Pluripotent neural crest stem cells in the adult hair follicle. Dev Dyn. 2004;231(2):258‐269. 15366003 10.1002/dvdy.20129

[20] Lothian C , Lendahl U . An evolutionarily conserved region in the second lntron of the human nestin gene directs gene Exmession to CNS progenitor cells and to early neural Ciest cells. Eur J Neurosci. 1997;9(3):452‐462. 9104587 10.1111/j.1460-9568.1997.tb01622.x

[21] John N , Cinelli P , Wegner M , Sommer L . Transforming growth factor β‐mediated Sox10 suppression controls mesenchymal progenitor generation in neural crest stem cells. Stem Cells. 2011;29(4):689‐699. 21308864 10.1002/stem.607

[22] Jones NC , Trainor PA . The therapeutic potential of stem cells in the treatment of craniofacial abnormalities. Expert Opin Biol Ther. 2004;4(5):645‐657. 15155156 10.1517/14712598.4.5.645

[23] Bressan RB , Melo FR , Almeida PA , et al. EGF–FGF2 stimulates the proliferation and improves the neuronal commitment of mouse epidermal neural crest stem cells (EPI‐NCSCs). Exp Cell Res. 2014;327(1):37‐47. 24907656 10.1016/j.yexcr.2014.05.020

[24] Jung Kim M , Cotman SL , Halfter W , Cole GJ . The heparan sulfate proteoglycan agrin modulates neurite outgrowth mediated by FGF‐2. Dev Neurobiol. 2003;55(3):261‐277. 10.1002/neu.10213 12717697

[25] Clewes O , Narytnyk A , Gillinder KR , Loughney AD , Murdoch AP , Sieber‐Blum M . Human epidermal neural crest stem cells (hEPI‐NCSC)—characterization and directed differentiation into osteocytes and melanocytes. Stem Cell Rev Rep. 2011;7(4):799‐814. 10.1007/s12015-011-9255-5 PMC3252033 21455606

[26] Horikiri T , Ohi H , Shibata M , et al. SOX10‐nano‐lantern reporter human iPS cells; a versatile tool for neural crest research. PLoS one. 2017;12(1):e0170342. 28107504 10.1371/journal.pone.0170342 PMC5249153

[27] Monsoro‐Burq A‐H , Fletcher RB , Harland RM . Neural crest induction by paraxial mesoderm in Xenopus embryos requires FGF signals. Development. 2003;130(14):3111‐3124. 12783784 10.1242/dev.00531

[28] Geary L , LaBonne C . FGF mediated MAPK and PI3K/Akt signals make distinct contributions to pluripotency and the establishment of neural crest. Elife. 2018;7:e33845. 29350613 10.7554/eLife.33845 PMC5790379

[29] Pegoraro C , Figueiredo AL , Maczkowiak F , Pouponnot C , Eychène A , Monsoro‐Burq AH . PFKFB4 controls embryonic patterning via Akt signalling independently of glycolysis. Nat Commun. 2015;6:5953. 25601028 10.1038/ncomms6953

[30] Rogers CD , Archer TC , Cunningham DD , Grammer TC , Casey EMS . Sox3 expression is maintained by FGF signaling and restricted to the neural plate by vent proteins in the Xenopus embryo. Dev Biol. 2008;313(1):307‐319. 18031719 10.1016/j.ydbio.2007.10.023 PMC2211421

[31] Tjaden NEB , Trainor PA . The developmental etiology and pathogenesis of Hirschsprung disease. Transl Res. 2013;162(1):1‐15. 23528997 10.1016/j.trsl.2013.03.001 PMC3691347

[32] Snider TN , Mishina Y . Cranial neural crest cell contribution to craniofacial formation, pathology, and future directions in tissue engineering. Birth Defects Res C Embryo Today. 2014;102(3):324‐332. 25227212 10.1002/bdrc.21075 PMC4320944

