# Supplementary material for: Derivation of neural crest stem cells from human epidermal keratinocytes requires FGF‐2, IGF‐1, and inhibition of TGF‐β1
Source: Bioeng Transl Med. 2018 Oct 1;3(3):256–64. doi: 10.1002/btm2.10109 (PMC6195909; doi:10.1002/btm2.10109)
Supplement: Supplementary file 1 — Supplementary Table 1 [file BTM2-3-256-s001.docx]

**Supplementary Table 1**

| Antibodies used | Dilution | Catalog # / Company |
| --- | --- | --- |
| Anti-Sox10 | 1:200 | #89356 / Cell Signaling |
| Anti-NES | 1:200 | #MAB5326 / EMD Millipore |
| Anti-FoxD3 | 1:200 | #2019 / Cell Signaling |
| Anti-Pax3 | 1:200 | **#**38-1801 Invitrogen |
| Anti-K14 | 1:400 | **#**PA5-28002 Invitrogen |
